# The Antigen Presenting Cells Instruct Plasma Cell Differentiation

**DOI:** 10.3389/fimmu.2013.00504

**Published:** 2014-01-06

**Authors:** Wei Xu, Jacques Banchereau

**Affiliations:** ^1^Pharma Research and Early Development, F. Hoffmann-La Roche Ltd., Roche Glycart AG, Schlieren, Switzerland; ^2^The Jackson Laboratory, Institute for Genomic Medicine, Farmington, CT, USA

**Keywords:** plasma cells, antigen presenting cells, macrophages, dendritic cells, B cells

## Abstract

The professional antigen presenting cells (APCs), including many subsets of dendritic cells and macrophages, not only mediate prompt but non-specific response against microbes, but also bridge the antigen-specific adaptive immune response through antigen presentation. In the latter, typically activated B cells acquire cognate signals from T helper cells in the germinal center of lymphoid follicles to differentiate into plasma cells (PCs), which generate protective antibodies. Recent advances have revealed that many APC subsets provide not only “signal 1” (the antigen), but also “signal 2” to directly instruct the differentiation process of PCs in a T-cell-independent manner. Herein, the different signals provided by these APC subsets to direct B cell proliferation, survival, class switching, and terminal differentiation are discussed. We furthermore propose that the next generation of vaccines for boosting antibody response could be designed by targeting APCs.

## Introduction

B cell activation is initiated following engagement of the B cell receptor (BCR) by a specific antigen in either a T-cell-dependent (TD) or T-cell-independent (TI) manner (1). Most long-lived plasma cells (PCs) in the bone marrow are derived from TD responses involving germinal center reactions followed by niches favoring long-term survival. As it usually takes several days for the cognate T cells to help, a prompt TI response provides the first wave of humoral protection by generating short-lived PCs in the extrafollicular foci of the peripheral lymphoid organs such as lymph nodes, spleen, Peyer’s patches, and tonsils (2). Indeed, some TI challenges could also induce long-lived antibody responses (3–5).

**Figure 1:**
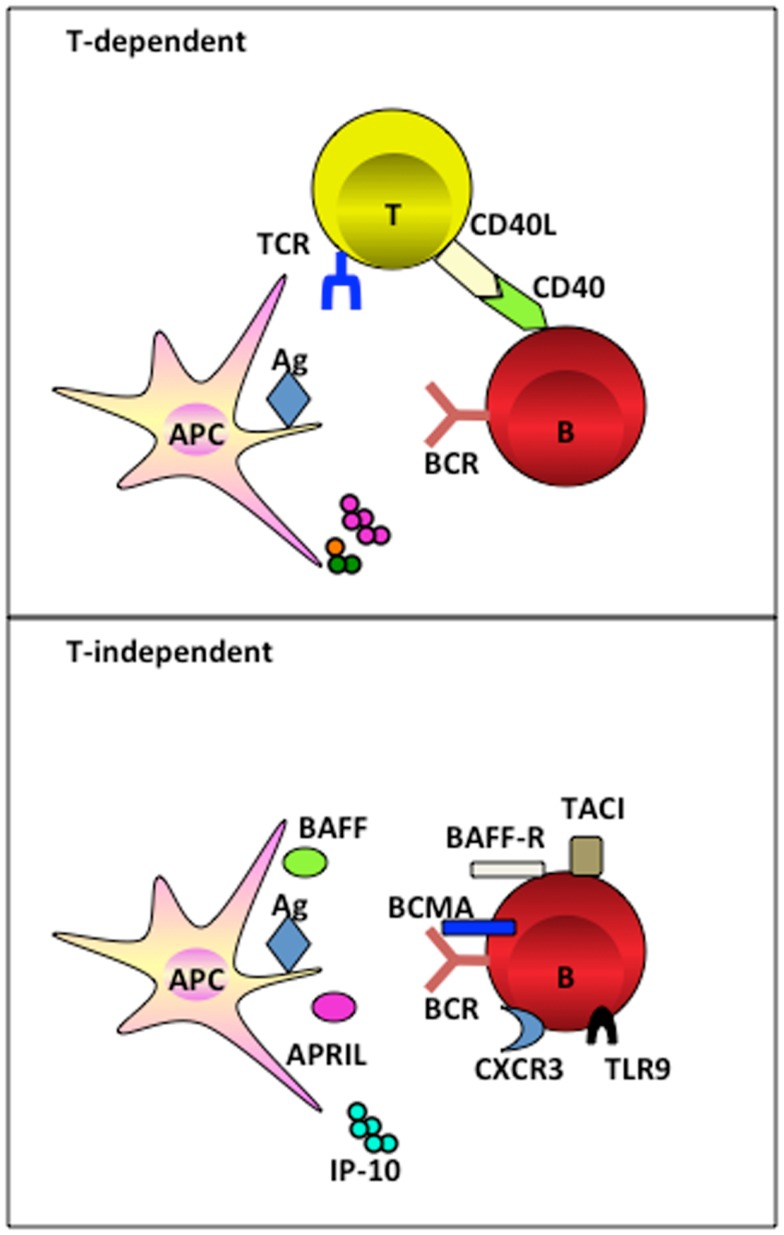
**APC subsets induce TI and TD B cell activation**. APC subsets including DCs and macrophages, have the capacity to retain and recycle native antigens on their surface and engage with B cell receptor for the signal 1 delivery. Ag-experienced B cells receive cognate T cell signals to form germinal center for the generation of long-lived PCs in a TD manner. Alternatively, APC-derived soluble factors provide late signals to B cells that have the respect receptors during the activation, proliferation, and differentiation, in a TI manner.

Professional antigen presenting cells (APCs), including dendritic cells (DCs) and macrophages, present antigens to T cells to initiate adaptive immunity by sequentially delivering signal 1 (antigen), signal 2 (co-stimulation), and signal 3 (polarizing signals mediated by soluble or membrane-bound factors) (6). They can, by similar means, initiate and guide B cell differentiation toward PCs in a TI manner. Precisely, DCs and macrophages efficiently take up large size antigens (such as particulates, immune complexes, and virus that travel through the subcapsular sinus), and present them to naïve B cells in the periphery lymphoid organs (2). Recent advances have revealed that APCs deliver not only signal 1, but also late signals to instruct terminal differentiation of PCs in both a TI and TD manner. In a TD manner, CD40-CD40L interaction between B cells and cognate T cells is instrumental in driving germinal center formation for affinity maturation. Whereas in a TI manner, APC-derived factors and the ligand-receptor signals between APC and B cells combines to deliver signals for PC differentiation (Figure 1). This review discusses the signals provided by these APC subsets and shapes a rationale of designing therapeutic vaccines for humoral immunity by targeting APCs.

## DC Subsets Instruct B Cell Differentiation

Back in the 1990s, following the early milestone discovery of DCs in mouse (7) and human (8), DCs have been recognized for their capacity of priming naïve B cells in human *in vitro* settings (9–11). In the presence of CD40 signaling, naïve B cells undergo class switching toward IgA1 and IgA2 isotype by DCs, and class switching (11). These early works using human monocyte-derived DCs provided the first evidence that in addition to their capacity to activate naive T cells in the extrafollicular areas of secondary lymphoid organs, DCs may directly modulate B cell growth and differentiation. Similarly, mouse splenic DCs were able to interact with naïve B cells and induce TI class switching *in vitro* and *in vivo* (12).

Dendritic cells directly induce TI Ab class switching through the upregulation of B lymphocyte stimulator protein (BLyS, also known as BAFF), and a proliferation-inducing ligand (APRIL) (13). BAFF binds to three different receptors, namely transmembrane activator and calcium modulator and cyclophylin ligand interactor (TACI), B cell maturation antigen (BCMA), and BAFF receptor (BAFF-R) (14–18). On the other hand, APRIL binds to BCMA with high affinity and to TACI with low affinity, but not to BAFF-R (19, 20). Through engagement with its receptors, BAFF activates a CD40-like pathway that enhances B cell survival via upregulation of NF-κB and Bcl-2 (21). APRIIL appears to induce AID expression in B cells through NF-κB-mediated HoxC4 induction (22). The importance of BAFF and APRIL has been documented in animal models where mice deficient for BAFF or APRIL showed a defect in IgA production (23, 24). Interestingly, B cells exposed to BAFF and APRIL do not secrete IgG and IgA unless stimulated through extensive BCR cross-linking. Thus, in a process of DC-mediated B cell differentiation, DCs initially provide TI antigens to engage BCR on B cells for activation. Thereafter, co-signals from other DC-derived factors like BAFF or APRIL or cytokines such as IL-15 cooperatively instruct the terminal differentiation of activated B cells into PCs (13).

Heterogeneous populations of DCs have been discovered in both human and mouse (25). In humans, three subsets have been identified in blood, namely CD303^+^ plasmacytoid DCs (pDCs), CD1c^-^CD141^+^, and CD1c^+^CD141^−^ circulating DCs (26–28). In the skin, cutaneous DCs express a distinct set of receptors as compared to blood DCs, i.e., langerin^+^ langerhans cells and CD14^+^ interstitial dermal DCs (29, 30). Among all subsets, interstitial dermal DCs that represent the *in vivo* counterpart of *in vitro* monocyte-derived DCs, appear to be the ones that preferentially prime B cells for humoral response while poorly triggering CD8^+^ T cell immunity (31), owing to their capacity to polarize follicular T help cells (Tfh) via DC-derived molecular such as IL-6 (32–34).

Plasmacytoid DCs, the professional type-1 interferon (IFN)-producing cells, promote the differentiation of CD40-stimulated B cells into non-antibody-secreting plasmablasts via IFN-αβ. They sequentially differentiate into antibody-secreting PCs upon additional IL-6 secreted by pDCs (35). Both B cells and pDCs express TLR9. IFN-α production by CpG ligation of the TLR9 on pDCs also generate IgM-producing PCs from both naïve and memory B cells in a TI manner, under the help of other pDC-derived factors such as IL-6, TNF-α, and IL-10 (36). TLR9 ligation of pDCs enhances their CD70 expression to trigger CD27 signaling for B cell survival and differentiation, particularly on memory cells (37). Type-1 IFN can also contribute to PC differentiation indirectly via the upregulation of BAFF and APRIL on myeloid DCs to promote B cell survival, proliferation, and class switching (38), or via promoting Tfh differentiation through myeloid DCs (39). In autoimmune disorders such as systemic lupus erythematosus (SLE), pDCs could be the driver favoring persistence of autoreactive PCs, giving the abnormal signature of type-1 IFN and autologous DNA and DNA-binding proteins (40–42). Indeed, activated pDCs trigger anti-snRNP B cells for enhanced proliferation and antibody production in the mouse (43).

How do B cells acquire antigens from DCs? DCs are found not only in the T cell areas of lymphoid organs where they are ready to prime T cells, but are also interacting with B cells in the follicular areas (44), the red pulp (45), and the marginal zones (46). DCs have a specialized capacity for the retention of antigens (44), enabling delivery of microbes from the intestinal lumen to secondary lymphoid structures (47, 48). Intravital two-photon imaging has revealed that upon lymph node entry, B cells physically survey local antigen-carrying DCs (49). DCs use different receptors to sample antigens that are directed to the degradative compartment for peptide and MHC loading. Interestingly, those antigens or immune complexes internalized by the inhibitory FcγRIIB on DCs were stored in a recycling versical system, largely excluded from the LAMP-1^+^ degradative compartment (50). As a consequence, these antigens were trapped in a native form, and recycled to the cell surfaces for the activation of B cells. This strategy for sorting and recycling native antigens through a non-degradative compartment is also used by follicular DCs to access B cells (51). Another inhibitory receptor, dendritic cell immunoreceptor (DCIR), holds the similar property as FcγRIIB for native antigen recycling utilized by marginal zone DCs to initiate B cell activation in a TD manner (52). It has been reported that even in the degradative late endosome, antigens can be released unprocessed by DCs (53). Thus DCs are equipped with an array of machinery to efficiently retain native antigens to BCR engagement on naïve B cells in a TI or TD manner.

## Macrophage Subsets Instruct B Cell Differentiation

Due to the nature of lymphoid structure, it has been conceived for a long time that lymph-born antigens must pass through a zone of macrophages that are beneath the subcapsular sinus *en route* to reach the follicular B cells (54–56). Macrophages are known to retain antigens for up to 72 h after being exposed to them (57). The very first evidence that macrophages process large size antigens (immune complexes, particulates, and viruses) to present to follicular B cells were found by three impendent groups (58–61). The subcapsular sinus macrophages possibly use CD169 or MAC1 (macrophage receptor 1) to retain antigens on their surface, and consequently B cells acquire antigens from them cumulatively and became the main antigen carriers inside the follicle before polarizing to the B cell-T cell border (58, 59). These studies clearly defined the essential roles of macrophage subsets in the initiation of B cell activation toward lymph-born antigens through dual actions: (1) as innate “flypaper” by preventing the systemic spread of pathogen; (2) as “gatekeepers” at the lymph-tissue interface that facilitate the recognition antigens by B cells and initiate humoral immune responses.

Macrophages residing in the marginal zone have the similar capacity to capture antigen in the spleen (62). Marginal zone macrophages (MZM) express a distinct set of receptors MARCO (macrophage receptor with a collagenous structure) and/or SIGNR1 (a mouse homolog of DC-SIGN), and are therefore different from metallophilic macrophages that express MOMA-1. The first study performed by Ravetch and his colleagues showed that MARCO^+^ MZM migrate to the red pulp of the spleen and transfer the intact antigens to B cells (63). It seems that SIGNR1 is important for the MZM-mediated B cell response, as MZM that lack expression of SIGNR1 failed to capture the model antigen Ficoll (64), and mice deficient for SIGNR1 failed to mount a humoral response following infection with *Streptococcus pneumoniae* (65).

**Figure 2:**
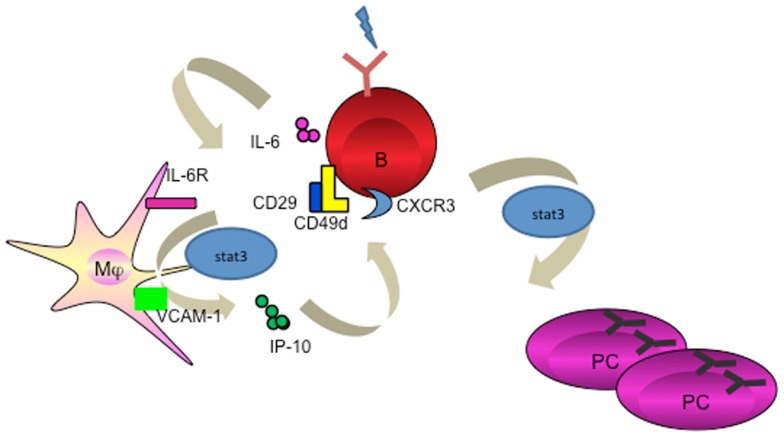
**Macrophage mediates PC differentiation**. Activated B cells release IL-6 to activate macrophage through STAT3. Macrophage-derived IP-10 sequentially binds to CXCR3 on B cells to trigger amplification loop production of IL-6, leading to STAT3-dependent PC differentiation.

In humans, the evidence for an exclusive role of macrophages in the induction of humoral response remains scarce. We recently identified that resident tissue macrophages in human tonsils reside closely to the terminally differentiated CD138^+^ PCs. We went on to unravel that macrophage-derived IP-10 participates in PC development (proliferation, class switching, and terminal differentiation) in the context of an amplification loop where B cell-derived IL-6 induces macrophages to secrete IP-10, which further boosts the B cell autocrine secretion of IL-6 leading to PC differentiation (Figure 2) (66). This is the first evidence that a chemokine plays direct role in cell differentiation. In addition, macrophages use VCAM-1 to tether B cells for the delivery of signals (66), supporting the earlier findings that VCAM-1 receptor-ligand interaction promotes membrane-bound antigen recognition and formation of an immune synapse (67).

Like DCs, macrophages promote TI class switching recombination by releasing the essential factors BAFF and APRIL (68–70). Macrophage-derived BAFF and APRIL expression can be enhanced by T cell signals such as IFN-γ and CD40L (68). B cell proliferation and antibody secretion following by BAFF and APRIL stimulation also requires co-stimulatory signals such as IL-6, IL-10, and TGF-β (13, 68, 70). This also implies that there are redundant signaling pathways involved in PC differentiation. For example, in rodents, subcapsular macrophages activate extrafollicular B cells indirectly through presenting CD1d-restricted glycolipid antigens to iNKT cells. PCs homing to the bone marrow require survival niches for long-term residence, and macrophages and their precursors provide such help through APRIL and IL-6 (71–73).

## Targeting APCs for a Better Vaccine for Humoral Immunity

**Figure 3:**
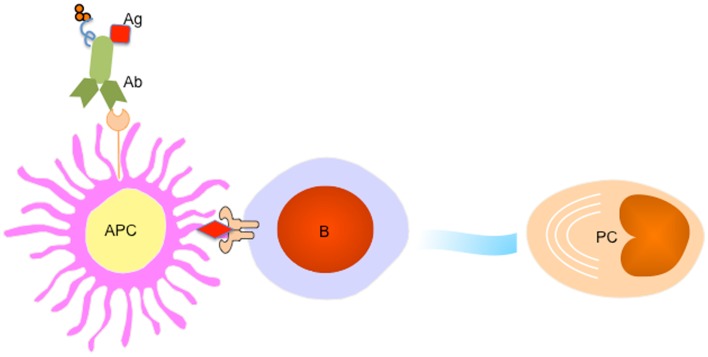
**Targeting APC subsets for a better vaccine for humoral immunity**. A fusion protein of Ab (recognizing a particular receptor on APC subset) and Ag complex facilitates Ag uptake by targeted APC subset, which processes Ag to B cells to trigger PC differentiation. An adjuvant (for example IP-10) could be linked to the fusion protein to provide additional signals for PC generation and maintenance.

Accumulating evidence suggest that APC subsets including DCs and macrophages not only provide “signal 1” for BCR engagement on B cells (74, 75), but further participate in a later stage of cell proliferation and differentiation by providing an additional “signal 2 or 3” such as membrane-bound or soluble factors. While interruption of this pathway might represent an efficient strategy to treat autoimmune diseases, enhancing APC-B cell crosstalk, for example by targeting Ag directly to APCs, may lead to enhanced vaccine-induced Ab responses (Figure 3).

Lessons of early pioneering studies *in vivo* targeting DCs through coupling the antigens to a specific receptors such as DEC-205, or DCIR for T cell immunity have paved a solid path toward understanding the efficiency of antigen degradation, and (cross-) presentation (76–78). Indeed, targeting antigens to DC through DCIR (79, 80), DC-SIGN (81), dectin-1 (82), ClEC9A (83), and Langerin (84) generated both humoral and cellular responses. Interestingly, in the absence of adjuvant, targeting antigens to CLEC9A on DCs results in strong antibody response, which is linked to the generation of Tfh cells (85), but no CD8^+^ T cell immunity despite of the antigen capture and cross-presentation by targeted CD8α^+^ DCs (83). However, an addition of adjuvant, e.g., poly I:C, skewed a robust CD4^+^ and CD8^+^ T cell response (83, 86). Thus, particular DC subsets, antibodies specific for surface receptors, and appropriate adjuvants, combine to define the sequential immune response by DC targeting (Table 1) (87).

**Table 1:** **Strategy to design a APC-targeted vaccine**.

Selection of APCs	Selection of targeting receptors	Selection of adjuvant
DCs	FcγRIIB	IP-10
	DCIR	IL-6
	DC-SIGN	APRIL
	Dectin-1	BAFF
	CLEC9A	
	Langerin	
	CD11c	
Macrophages	CD163	
	FcγRIIB	

*To design the appropriate targeted vaccine, three criteria need to be considered: (1) Select the appropriate DC or macrophage subsets as the targeting APCs; (2) Select appropriate receptor to target, preferentially those receptors with capacity of Ag recycling and retention, such as DCIR or FcγRIIB; (3) Select appropriate adjuvant to provide additional help for PC differentiation*.

The strategy of a targeted DC vaccine with an antigen to boost antibody response has met the proof of concept. In two of the studies, targeting DCs through CD11c (N418) showed robust humoral immunity resulting from germinal center formation (88, 89), though mechanistic details about antigen internalization and transfer and the factors involved in PC generation by DCs were lacking. Likely, two principles must be followed to design a better vaccine to boost Ab response by targeting DCs in humans; (1) preferentially target interstitial dermal DCs due to their capacity to activate B cells (31); (2) preferentially deliver antigens through inhibitory receptors such as FcγRIIB (50) or DCIR (52) to enable long-term retention and recycling of native antigens to the cell surfaces. The selection of adjuvant would be based on whether it needs to promote B cell differentiation (such as BAFF and APRIL), or it needs to educate Tfh cells (such as IL-6).

Our study on human macrophages in the induction of PCs (66) suggests that targeting CD163 on resident tissue macrophages would be another approach to potentially trigger preferred antibody response. IP-10 may act as a powerful adjuvant to provide the feedback loop for IL-6 production on activated B cells. Using systems biology approach, we observed that IP-10 signature was quickly turned on after influenza vaccination in healthy individuals, and it was corresponding to the late neutralizing antibody and PC signature (90). Mice deficient for IP-10 showed reduced antibody titers against the model antigen hapten, further supporting the wide application of this macrophage-derived molecule in vaccine design (66).

Of note, targeting antigens to different subsets of APCs could lead to a differential class switching. Our preliminary data indicate that among the myeloid APCs generated from monocytes, DCs preferentially induce IgG-producing cells, whereas type 2 macrophages (M2) preferentially promote IgA-producing cells (Xu et al., unpublished). The mechanism of APC subset-mediated preferential class switching remains to be explored further. It will lead to a better understanding of vaccine design when a unique Ig subclass response is needed.

## Concluding Remarks

The past decade has witnessed the important roles of DCs and macrophages in educating B cell activation, proliferation, and differentiation toward PCs. These APC subsets residing at distinct organs might be equipped different sentinels to initiate the prompt humoral response. For example, the subcapsular sinus macrophages, which form a thick lining beneath the capsular in the lymph node, represent the prime APCs to deliver combined signals to naïve B cells for priming. As compared to DC-targeted vaccines for T cell immunity that are applied for more than a decade (91), we are just beginning to design APC-targeted vaccines aiming at enhance antibody responses. As such, various studies have helped our understandings that the interplay of several distinct factors needs to be considered (1) selection of APC subsets as the target cells; (2) selection of appropriate surface receptors as the antibody target; (3) selection of adjuvant.

## Conflict of Interest Statement

The authors declare that the research was conducted in the absence of any commercial or financial relationships that could be construed as a potential conflict of interest.
